# Extended-spectrum beta-lactamases in clinical isolates of *Escherichia coli* and *Klebsiella pneumoniae* recovered from patients at the Tamale Teaching Hospital, Ghana

**DOI:** 10.1371/journal.pone.0300596

**Published:** 2024-04-05

**Authors:** Francis Kwame Morgan Tetteh, Anthony Ablordey, Noah Obeng-Nkrumah, Japheth Awuletey Opintan

**Affiliations:** 1 Department of Medical Microbiology, University of Ghana Medical School, Accra, Ghana; 2 Pathology Division, 37 Military Hospital, Accra, Ghana; 3 Department of Bacteriology, Noguchi Memorial Institute for Medical Research, University of Ghana, Accra, Ghana; North Carolina State University, UNITED STATES

## Abstract

**Introduction:**

Extended-spectrum beta-lactamase (ESBL)-producing *Escherichia coli* and *Klebsiella pneumoniae* are pathogens of significant public health interest for which new antibiotics are urgently needed.

**Aim:**

To determine the prevalence of ESBLs in *E*. *coli* and *K*. *pneumoniae* isolates from patients attending the Tamale Teaching Hospital (TTH) in Ghana.

**Methodology:**

The study was a cross-sectional study involving convenience sampling of *E*. *coli* and *K*. *pneumoniae* isolates from consenting patients’ clinical specimens, between April and June 2015. Antimicrobial susceptibility test was performed, and ESBL-producer phenotypes were further screened for *Bla*_TEM_, *Bla*_SHV_, and *Bla*_CTX-M_ genes. Patients’ clinical data were additionally collected using a structured questionnaire.

**Results:**

Of the 150 non-duplicate *E*. *coli* and *K*. *pneumoniae* isolates identified, 140 were confirmed as *E*. *coli* (84%, *n* = 117) and *K*. *pneumoniae* (16%, *n* = 23). Of these, sixty-two (44%) [*E*. *coli* (84%; *n* = 52); *K*. *pneumoniae* (16%; *n* = 10)] phenotypically expressed ESBLs. The proportion of ESBL-producing isolates was higher in adults (15–65 years) than in neonates (< 28 days) (*p =* 0.14). Most of the isolates showed a high percentage resistance to ampicillin (96%) and tetracycline (89%), but a relatively lower resistance to amikacin (36%). No isolate was resistant to meropenem. More ESBL producers were multidrug resistant compared to non-ESBL-producers [23% (14/62) versus 18% (14/78); *p* = 0.573]. Overall, 74% (*n* = 46) of the ESBL genotypes expressed *Bla*_CTX-M-1_ genes, followed by 63% (*n* = 39) *Bla*_TEM_, and 16% (*n* = 10) *Bla*_SHV_. The study showed a high prevalence of ESBL-positive *E*. *coli* and *K*. *pneumoniae*, mostly CTX-M-1 producers at TTH.

**Conclusion:**

Routine laboratory ESBL screening is warranted to inform patient management.

## Introduction

Βeta-lactamases are bacterial enzymes that inactivate beta-lactam antibiotics by hydrolysis [1], and cause resistance to penicillins, cephalosporins, and monobactams, but not cephamycins or carbapenems [2]. Among the groups of beta-lactamase enzymes, extended-spectrum beta-lactamases (ESBLs), Class C cephalosporinases (AmpCs) and carbapenemases constitute the chief resistance determinants against beta-lactam antibiotics. Of these, ESBLs are the commonest source of resistance to the beta-lactams [3].

The spread of bacterial resistance is mainly mediated by plasmids, as they can be transferred between Gram-negative bacteria by conjugation [4]. This transferability is responsible for outbreaks of resistance [5, 6]. ESBLs include the widespread plasmid-encoded enzyme families and their variants: Temoniera (TEM) [7], Sulfhydryl variable (SHV), and Oxacillinases (OXA) [5]. In recent years, many ESBLs of non-TEM, non-SHV and non-OXA types, especially the cefotaximase (CTX-M), have been detected and reported worldwide [5]. The CTX-M type ESBL spreads rapidly, and is now one of the most dominant types of ESBL in many countries [8, 9]. *Escherichia coli* and *Klebsiella pneumoniae* remain the major ESBL-producing organisms isolated worldwide, but these enzymes have also been identified in several other members of the *Enterobacteriaceae* and in some non-fermenting Gram-negative bacteria like *Pseudomonas spp*. and *Proteus mirabilis* [3].

ESBL-producing enterobacteria have spread across the world, but some health authorities have limited awareness of this problem, especially in African countries [10]. In many African countries, the issue of routine screening for ESBLs remains contentious, owing to the huge financial demands involved [10]. In Ghana, most clinical laboratories do not routinely screen for ESBLs [11]. Documented surveys exist on resistance of *Enterobacteriaceae* in Korle-Bu Teaching Hospital (KBTH) [12] and Komfo-Anokye Teaching Hospital (KATH) [13] to extended-spectrum cephalosporin resistance and resistance to other non-beta-lactam antibiotics. Recent findings from a study on the geographical distribution of beta-lactam resistance among *Klebsiella* spp. from selected health facilities across Ghana revealed worrying levels of beta-lactam resistance among *Klebsiella* spp. within the northern sector compared to the middle and southern sectors [14]. Nonetheless, published reports on ESBL-producing isolates from the Tamale Teaching Hospital (TTH), which provides medical services to the northern sector of Ghana, seems limited. It is, therefore, imperative to heighten awareness among clinicians and policy-makers, and enhance testing by clinical laboratories, for the detection of ESBLs. To facilitate such efforts, this study determined the prevalence of ESBLs in clinical isolates of *E*. *coli* and *K*. *pneumoniae* from patients attending TTH and characterized the encoding genes.

## Materials and methods

### Study design and setting

This cross-sectional study was carried out in Ghana, a country located in West Africa along the Gulf of Guinea and the Atlantic Ocean [15] and has a population of approximately 32 million [16]. Healthcare in the country is largely administered by the Ministry of Health and the Ghana Health Service [17]. The country has instituted a national universal medical insurance system, the National Health Insurance Scheme (NHIS), which covers basic inpatient and primary care outpatient services for patients who purchase the insurance [17]. However, for those who do not enroll in the NHIS, they are left to pay for all services and medications out-of-pocket [18].

The study involved convenience sampling of *E*. *coli* and *K*. *pneumoniae* from consenting patients’ clinical specimens through verbal interaction at the TTH. The verbal interaction involved two key questions to the patients, that is, whether previous antibiotics medications were prescribed by a doctor or self-prescribed, and whether they completed the previous medication. We reviewed patients’ antibiotic usage history from their hospital folders whenever possible. When folders were unavailable, we focused on patients’ self-reported antibiotic use within the past three months to minimize the impact of recall bias. TTH is in the eastern part of the Tamale Metropolis, a catchment area which has a population of approximately 2.1 million. The hospital was established to serve as a medical referral centre for the northern sector of Ghana and some neighboring countries, including La Cote D’Ivoire, Burkina Faso, and Togo. It has a bed capacity of four hundred and fifty-two (452) and a microbiological laboratory that provides routine bacteriological services to the hospital and the population in general, and processes over 8,000 clinical cultures annually [19].

### Isolate collection and transportation

During three months, April–June 2015, 4,850 clinical samples from 4,100 unique patients were submitted to the microbiology laboratory of the TTH for bacteriologic investigations. The specific specimens for bacteriological investigations were collected based on physicians’ clinical diagnosis and the requested investigation. From these clinical specimens, all *E*. *coli* and *K*. *pneumoniae* isolates cultured as aetiologic agents of clinical infection for which laboratory investigations were requested were prospectively included in the study. These recovered isolates were stocked on Mueller Hinton slants in cryovials and transported to our research laboratory at the Department of Medical Microbiology, College of Health Sciences, Korle Bu Campus, Accra, for confirmation and further analyses.

#### Isolate identity confirmation

The isolates were sub-cultured onto MacConkey agar plates and incubated in ambient air at 35 ± 2°C for 16 to 18 hours. A biochemical procedure using MINIBACT-E micro-test kits (SSI, Denmark) was then used to ascertain the identities of the isolates; 140 (117 *E*. *coli* and 23 *K*. *pneumoniae*) of the originally 150 clinical isolates were confirmed to be *E*. *coli* and *K*. *pneumoniae*. The isolates were stored in trypticase soy broth containing 10% glycerol at -20°C, until further workup.

### Antibiogram determination

Susceptibilities of isolates to the following antibiotics were assessed: ampicillin (10 μg), cefuroxime (30 μg), cefotaxime (30 μg), meropenem (10 μg), tetracycline (30 μg), chloramphenicol (30 μg), gentamicin (10 μg), amikacin (30 μg), ciprofloxacin (5 μg) and nalidixic acid (30 μg) (Oxoid, Basingstoke Hampshire, UK). These were determined using agar disk diffusion according to the Clinical and Laboratory Standard Institute (CLSI, 32nd edition) reference guidelines and breakpoints. Reference strains *E*. *coli* ATCC 25922 and *K*. *pneumoniae* ATCC 700603 were included as quality controls in each susceptibility test.

### Phenotypic determination of ESBL-producing isolates

#### ESBL screening

Recovered isolates were screened for presumptive presence of ESBLs using cefotaxime (30 μg), ceftazidime (30 μg) and cefpodoxime (10 μg) antibiotic disks (MAST Group Ltd., Bootle, UK) according to the CLSI guidelines [20]. Antibiotic disk diffusion tests were performed using 0.5 McFarland standard inoculums on Mueller Hinton agar (BioMerieux) and incubated overnight at 37°C. Using CLSI screening guidelines, bacterial isolates with zone inhibition diameters ≤ 27 mm for cefotaxime and ≤ 22 mm for ceftazidime were reported as positive for ESBL screening. As recommended by CLSI, cefpodoxime disks were included in the ESBL screening with breakpoints of ≤17 mm for study isolates. Study isolates resistant at these breakpoints were reported as positive for ESBL screening. All isolates with a positive result in the ESBL screening test with at least one of the three screening agents were selected for ESBL confirmation.

#### ESBL confirmation

Screened-positive bacterial isolates were confirmed for ESBL production using the combined-disk method according to the CLSI guidelines [20]. Zones of inhibition were determined for each isolate to antibiotic disks containing 30 μg of cefotaxime, 30 μg of ceftazidime and 10 μg of cefpodozime either alone or in combination with 10μg of clavulanic acid (MAST Group Ltd.). All zones of inhibition which differed by ≥ 5 mm between at least one of the standard antibiotic disks and its corresponding clavulanic combination disks were classified as having an ESBL-producer phenotype. *Escherichia coli* control strain ATCC 25922 was used to monitor the performance of ESBL detection agents.

### Molecular characterization of ESBL-producing isolates

Phenotypically documented ESBL-producing *E*. *coli* and *K*. *pneumoniae* were further characterized using conventional polymerase chain reaction (PCR) to confirm the presence of gene families that encoded ESBLs (TEM, SHV and CTX-M) after deoxyribonucleic acid (DNA) template extraction using Qiagen extraction kits (Qiagen, UK) at the Virology Department, Noguchi Memorial Institute for Medical Research (NMIMR), Accra–Ghana. The DNA concentrations and purities were quantified using a Nanodrop device (Thermo Scientific, USA). Isolates were screened for TEM, SHV and CTX-M-1, 2 and 9 cluster groups of ESBLs. PCR was carried out using thermal cycler (BioRad, USA) with a total volume of 25.0 μl containing 2.0 μl DNA template and 23.0 μl Master Mix (Qiagen, UK). The PCR products for each reaction were electrophoresized on 2% agarose gel with a 100-base pair molecular DNA marker (Biolabs, New England). Gels were visualized using the UV-transilluminator after staining in ethidium bromide. The presence of single bands of *Bla*_TEM_, *Bla*_CTX-M-1_ and *Bla*_SHV_ genes were observed on the gels at the expected band sizes of 918, 940 and 842 bp, respectively.

### Data analysis

Data were stored in Microsoft Excel and later exported to Statistical Package for Social Sciences, Version 16 (Atlanta, USA), for analysis. Point estimates of statistical significance were indicated with a 2-tailed test at *p*-values < 0.05. Descriptive statistics (frequencies and cross-tabulations) were used to determine the prevalence of ESBLs. Categorical data were compared across study parameters using the Chi square or the Fisher’s exact test, where appropriate. Univariate analysis was performed for each patient category to identify potential associations between respondents infected with ESBL-producing Enterobacterales and various independent predictor variables. The strength of these associations was quantified using unadjusted odds ratios (ORs) with 95% confidence intervals (CIs). Variables with a p-value <0.05 in the univariate analysis were subsequently included in multivariable logistic regression models to determine independent risk factors for ESBL-producing Enterobacterales infection. However, the Hosmer-Lemeshow goodness-of-fit test indicated that the multivariable logistic regression models did not predict the outcome accurately on average (p-value < 0.01). Additionally, the area under the receiver operating characteristic (ROC) curve was less than 0.2, suggesting that the models had poor discriminatory capability.

### Ethical approval

The Ethical and Protocol Review Committee (EPRC) of the College of Health Sciences, University of Ghana, approved the research study (protocol identification number: MS-Et/M.7-P3.2/2014-2015). Approval was also obtained from TTH. Written informed consent was obtained from all participants/next of kin before enrollment into the study and also for the collection of isolates and their subsequent processing. The study prioritized confidentiality by conducting interviews in private and confidential settings using trained nursing assistants. The collected information were coded to anonymize patient data and dissociate individuals’ identities from the study results. Participants’ records and laboratory data were kept under lock and key, with access restricted only to authorized personnel.

## Results

### General characteristics of the study participants

Out of 150 non-duplicate *E*. *coli* and *K*. *pneumoniae* isolates identified as the causative agents of infections from TTH, 140 were confirmed to be *E*. *coli* (n = 117, 84%) and *K*. *pneumoniae* (n = 23, 16%) in our research laboratory at the Department of Medical Microbiology, Korle Bu, Accra. Each of the 140 isolates recovered belonged to a single specimen and a different patient. The isolates were mainly from cultures of urine (56%; *n* = 78), HVS/ endocervical swabs (17%; *n* = 24), pus (9%; *n* = 12), wounds (7%; *n* = 10), sputum (6%; *n* = 9), and blood (5%; *n* = 7) (Table 1). The mean age of the patients was 32.7 ± 19.2 years, and comprised 44 males (mean age = 40.8 ± 22.3 years) and 96 females (mean age = 29.0 ± 16.4 years). Of the 117 patients infected with *E*. *coli*, 32% (*n* = 37) were males, and 68% (*n* = 80) were females. Also, of the 23 patients infected with *K*. *pneumoniae*, 30% (*n* = 7) were males and 70% (*n* = 16) were females.

**Table 1:** Logistic regression analysis of patient’s characteristics in relation to extended spectrum beta-lactamase in Tamale Teaching Hospital, Ghana (April—June 2015).

Variables	Total (*n* = 140)	ESBL		Odds ratio (95% CI)	*P-value*		
		Producers (*n* = 62)	Non-producers (*n* = 78)			Adjusted OR	P-value
**Socio-demographic data**							
**Age group**							
≤28d-5y		2 (3.2%)	4 (5.1%)	0 (0–0)	1.46		
>5y-15y		4 (6.5%)	1 (1.3%)	5.03 (0.55–46.18)	0.18	-	
>15y-65y		49 (79.0%)	65 (83.3%)	0.95 (0.58–1.56)	0.9	-	
>65y		7 (11.3%)	8 (10.3%)	1.10 (0.38–3.20)	1	-	
**Sex**							
Male		18 (29.0%)	26 (33.3%)	0.82 (0.40–1.69)	0.71	-	
Female		44 (71.0%)	52 (66.7%)				
**Diagnosis queried by Clinician**							
Wound abscess		5 (8.1%)	5 (6.4%)	1.30 (0.36–4.70)	0.75	-	
Sepsis		3 (4.8%)	4 (5.1%)	0.94 (0.20–2.23)	1	-	
Chest pain		0 (0.0%)	2 (2.6%)	0.00 (0.00–0.00)	0.5	-	
PID		3 (4.8%)	1 (1.3%)	3.92 (0.40–38.60)	0.32	-	
BPH		2 (2.3%)	3 (3.8%)	0.83 (0.13–5.15)	1	-	
Chronic cough		3 (4.8%)	4 (5.1%)	0.94 (0.20–4.37)	1	-	
Peritonitis		7 (11.3%)	3 (3.8%)	3.18 (1.05–12.86)	0.11	-	
Ascites		1 (1.6%)	1 (1.3%)	1.26 (0.08–20.60)	1	-	
Lower abdominal pain		1 (1.6%)	1 (1.3%)	1.26 (0.08–20.60)	1	-	
UTI/ painful urination		32 (51.6%)	41 (52.6%)	0.96 (0.49–0.88)	1	-	
Vaginal discharge		5 (8.1%)	13 (16.7%)	0.44 (0.15–1.31)	0.2	-	
**Sample type**							
Urine		34 (54.8%)	44 (56.4%)	0.97 (0.56–1.70)	1		
HVS		9 (14.5%)	15 (19.2%	0.75 (0.31–1.84)	0.66		
Pus		8 (12.9%)	4 (5.1%)	2.52 (0.72–8.74)	0.23		
Wound		5 (8.1%)	5 (6.4%)	1.26 (0.35–4.54)	0.75		
Sputum		3 (4.8%)	6 (7.7%)	0.63 (0.15–2.62)	0.73		
Blood		3 (4.8%)	4 (5.1%)	0.94 (0.20–4.37)	1		
**Previous antibiotics medication**							
• In past three months • Prescribed by doctor		62 (100%) 53 (85.5%)	78 (100%) 47 (60.3%)	3.88 (1.68–8.99)	1 *0.0008	2.90 (0.61–3.61)	3.55
• Self-prescribed		9 (14.5%)	31 (39.7%)				
**Completion of previous medication**		49 (79.0%)	50 (64.1%)	2.11 (0.98–4.54)	*0.0399	3.09 (0.11–2.86)	4.90

* Shows significant association in relation to ESBLs.

CI-Confidence interval, PID-Pelvic inflammatory disease, BPH-Benign prostatic hyperplasia, UTI-Urinary tract infection, HVS- High vaginal swab; clinically relevant age-groupings: ≤28d, neonates; 29d-1y, infants; >1y-5y, paediatrics (≤28d-5y, paediatrics); >5y-15y, children; >15y-65y, adults; >65y, elderly; The multivariable logistic regression produced models with poor discriminatory and predictive accuracy and the analysis were insufficient to determine risk factors for ESBL infections.

### Antibiotic susceptibility patterns of the isolates

Generally, there was a high percentage resistance of isolates to ampicillin (96%) and tetracycline (89%). Resistance to nalidixic acid and ciprofloxacin was at 77% and 74%, respectively. The isolates’ resistance to chloramphenicol, cefuroxime, gentamicin and cefotaxime were 60%, 56%, 54%, and 46%, respectively. A relatively lower resistance was observed for amikacin (36%). None of the isolates were resistant to meropenem.

### Phenotypic expression of ESBLs

Among the 140 study isolates, 50% (70 isolates) were resistant to at least one of the following cephalosporins: cefotaxime (30 μg), ceftazidime (30 μg), or cefpodoxime (10 μg). These isolates were considered screen positive for ESBLs and were further tested for confirmation. Sixty-two of the 70 clinical isolates were confirmed as ESBL-producers, corresponding to an ESBL-prevalence of 44% (n = 62/140). Of these, 84% (*n* = 52) were *E*. *coli* and 16% (*n* = 10) were *K*. *pneumoniae* isolates. The ESBL-producers were most prevalent in pus (n = 8/12, 67%), followed by HVS (n = 9/15, 60%), wound (n = 5/10, 50%), and urine (n = 34/78, 44%) (Table 2).

**Table 2:** Occurrence of ESBL-producing *E*. *coli* and *K*. *pneumoniae* across specimen type in Tamale Teaching Hospital, Ghana (April—June 2015).

Sample type	Number tested	ESBL-phenotype		ESBL-producing isolates	
		Screen positive (%)	ESBL-producers (%)	*E*. *coli* (%)	*K*. *pneumoniae* (%)
Urine	78	78 (100)	34 (44)	31 (91)	3 (9)
HVS	24	24 (100)	9 (38)	8 (89)	1 (11)
Pus	12	12 (100)	8 (67)	7 (87.5)	1 (12.5)
Wound	10	10 (100)	5 (50)	3 (60)	2 (40)
Sputum	9	9 (100)	3 (33)	1 (33)	2 (67)
Blood	7	7 (100)	3 (43)	2 (67)	1 (33)
Total	140	70 (50%)	62 (44%)	52 (84%)	10 (16%)

HVS- High vaginal swab.

The proportion of ESBL-producing isolates was relatively higher in adults (15–65 years) than in neonates (< 28 days), albeit not statistically significant (*p =* 0.14). However, age had no significant association with respect to ESBL-producing Enterobacteria (*p =* 0.14). Univariate analysis using patient’s characteristics and their association with ESBLs was performed (Table 1). Diagnosis and gender showed no significant association with ESBL-producing enterobacteria. All patients included in the study had received antibiotics in the past three months. Patients who reported their previous medication as having been prescribed by a physician as well as patients who completed their previous medication showed significant associations with ESBL-producing enterobacteria (*p* = 0.0008 and 0.0399 respectively).

### Antimicrobial resistance among the ESBL-producers and non-ESBL producers

**Fig 1:**
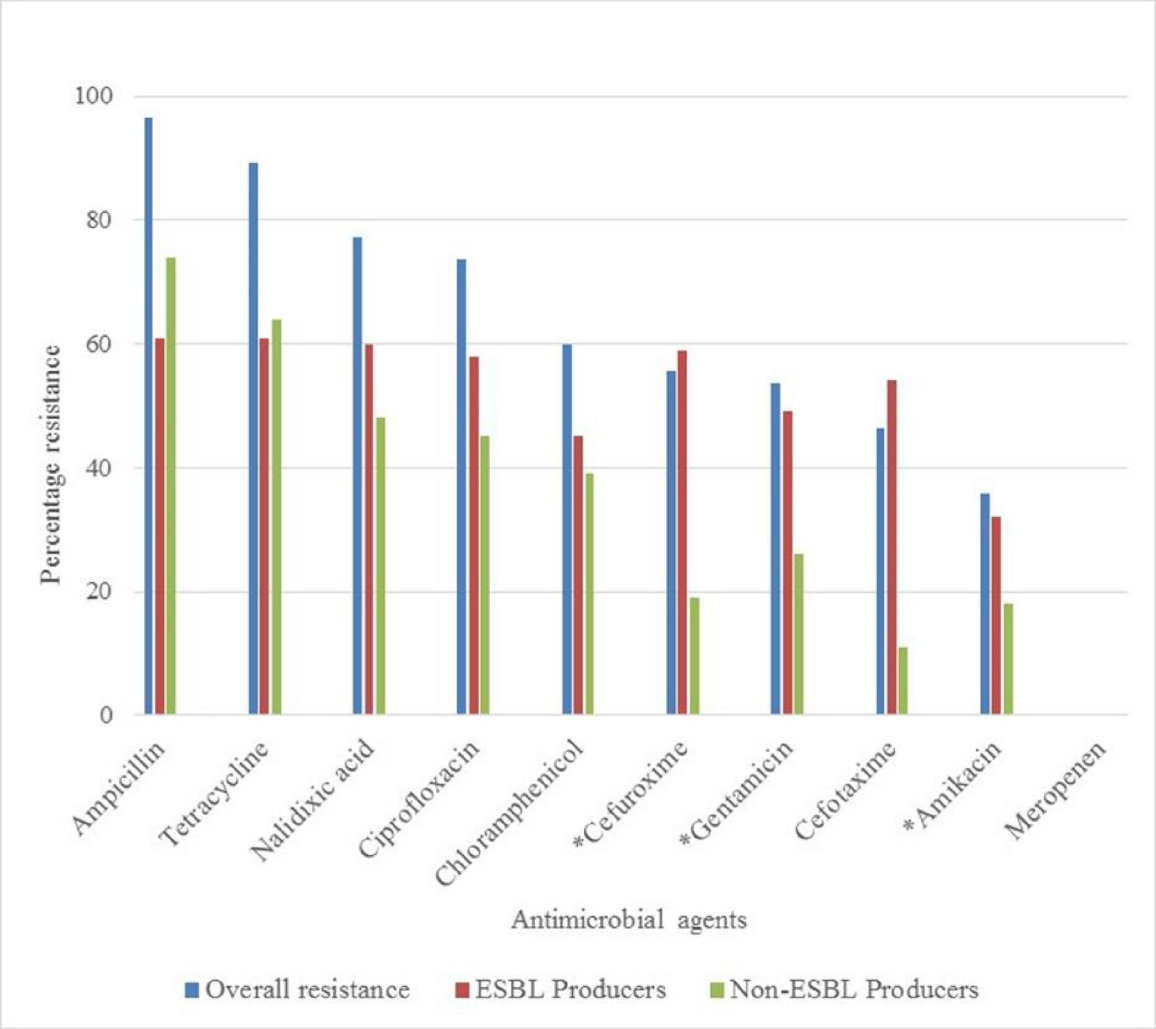
Antibiogram of ESBL-producers and non-ESBL producers in Tamale Teaching Hospital, Ghana (April—June 2015). In the figure, * = Antimicrobial resistance showing significant differences between ESBL-producers and non-ESBL-producers.

Comparison of antibiograms of ESBL producers and non-ESBL producers using the disk diffusion method of sensitivity testing was performed (Fig 1). Significant differences in antibiotic resistance between ESBL producers and non-ESBL producers were observed for cefuroxime (*p* < 0.001), gentamicin (*p* = 0.004) and amikacin (*p* = 0.02). However, all the isolates used in this study were susceptible to meropenem. Of the ESBL producers, 23% (*n* = 14) were multidrug resistant (MDR) strains, whereas 18% (*n* = 14) non-ESBL producers were observed to be MDR (Table 3).

**Table 3:** Enterobacteria expressing multidrug resistant phenotypes to amikacin, ciprofloxacin, tetracycline and cotrimoxazole in Tamale Teaching Hospital, Ghana (April—June 2015).

Antimicrobial drugs combinations	Multidrug resistant phenotypes	
	ESBL-producers (*n* = 62)	Non-ESBL-producers (*n* = 78)
AMK CIP TET	14 (22.6%)	14 (17.9%)
AMK CIP TET C	12 (19.4%)	8 (10.3%)

* AMK-Amikacin, CIP-Ciprofloxacin, TET-Tetracycline, C-Chloramphenicol.

### Detection of ESBL genotypes

**Fig 2:**
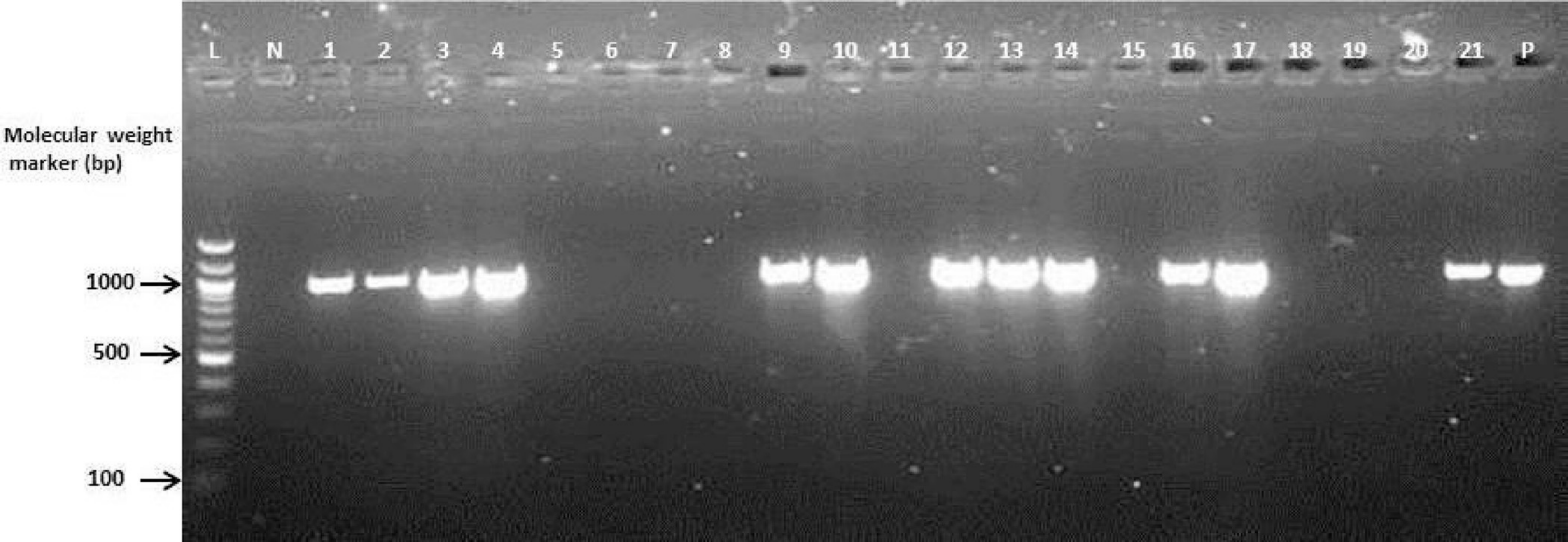
Gel electrogram targeting *Bla*_TEM_ gene of enterobacteria isolates in Tamale Teaching Hospital, Ghana (April—June 2015). The presence of *Bla*_TEM_ genes were observed with single bands at the expected band size of 918 bp. Lanes 1–4, 9, 10, 12–14, 16, 17 and 21 were positive for *Bla*_TEM_ gene. Lanes 5–8, 11, 15 and 18–20 were negative for *Bla*_TEM_ gene. Lanes L, N and P represents 100 bp molecular weight marker, negative and positive controls respectively.

**Fig 3:**
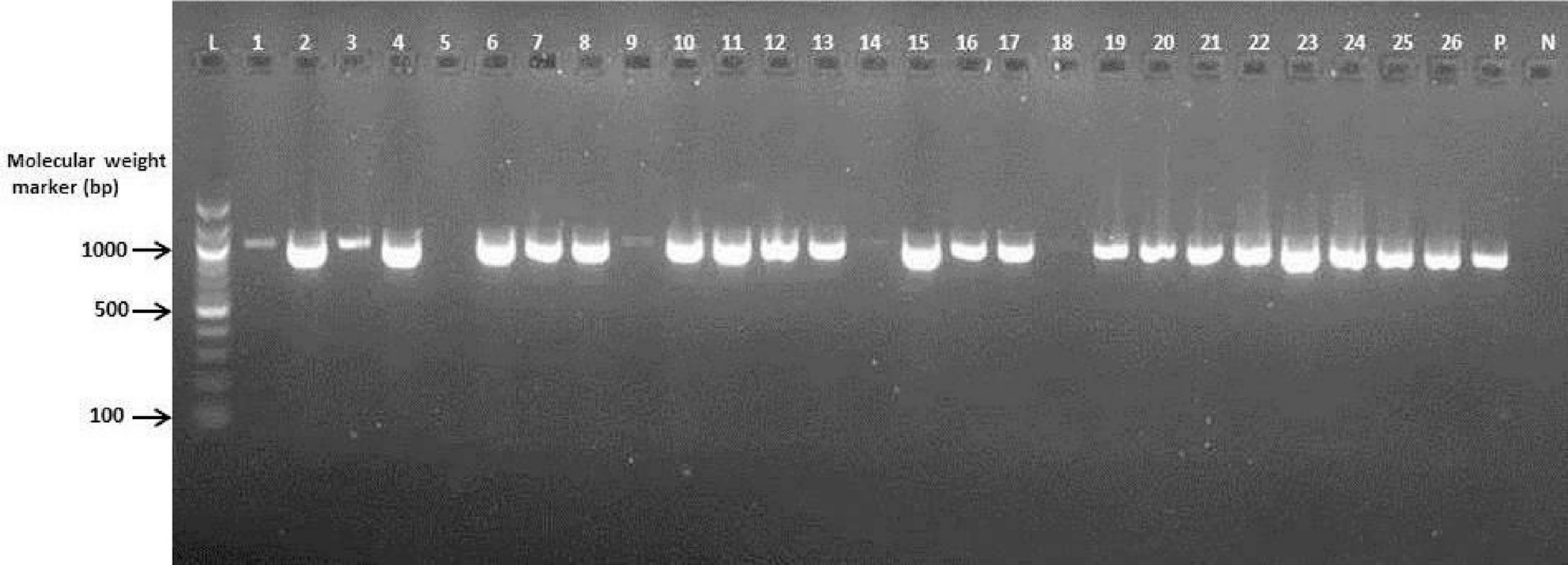
Gel electrogram targeting *Bla*_CTX-M-1_ gene of enterobacteria isolates in Tamale Teaching Hospital, Ghana (April—June 2015). The presence of *Bla*_CTX-M-1_ genes were observed with single bands at the expected band size of 940 bp. Lanes 1–4 and 6–26 were positive for *Bla*_CTX-M-1_ gene. Lanes 5, 14 and 18 were negative for *Bla*_CTX-M-1_ gene. Lanes L, N and P represents 100 bp molecular weight marker, negative and positive controls respectively.

**Fig 4:**
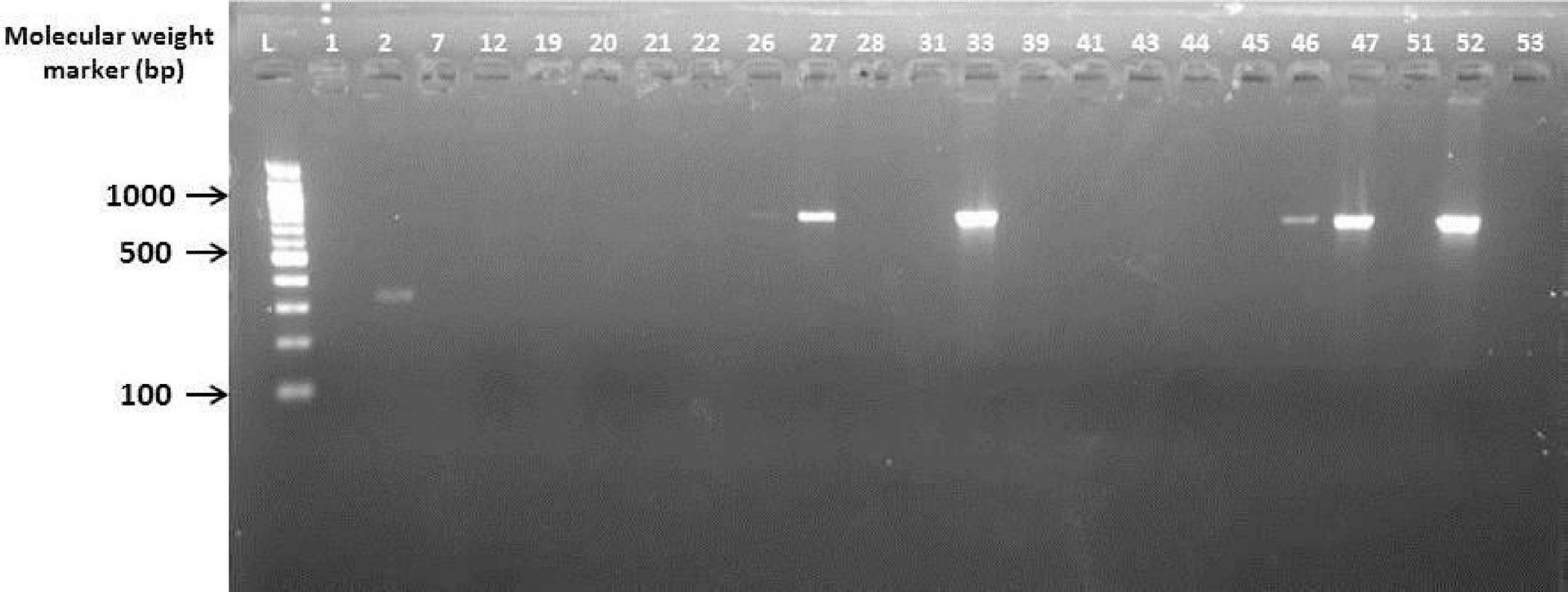
Gel electrogram targeting *Bla*_SHV_ gene of enterobacteria isolates in Tamale Teaching Hospital, Ghana (April—June 2015). The presence of *Bla*_SHV_ genes were observed with single bands at the expected band size of 842 bp. Lanes 26, 27, 33, 46 and 47 were positive for *Bla*_SHV_ gene. Lanes 1, 2, 7, 12, 19, 20, 21, 22, 28, 31, 39, 41, 43, 44, 45 and 51 were negative for *Bla*_SHV_ gene. Lanes L, 52 and 53 represents 100 bp molecular weight marker, positive and negative controls respectively.

The gel electrogram showed 12, 24, and 5 single bands of *Bla*_TEM_, *Bla*_CTX-M-1_, and *Bla*_SHV_ genes, respectively (Figs 2–4). About 74% of the ESBL genotypes expressed *Bla*_CTX-M-1_ genes, followed by 63% *Bla*_TEM_ and 16% *Bla*_SHV_. None of the isolates expressed genes for CTX-M 2 and CTX-M 9. About 6% (*n* = 4) of the isolates harbored all three genes (*Bla*_TEM_, *Bla*_SHV_ and *Bla*_CTX-M 1_). Overall, 13% (*n* = 8) of the isolates that phenotypically expressed ESBLs did not harbor any identifiable beta-lactamase genes (Table 4).

**Table 4:** Extended spectrum beta-lactamase genotypes in *E*. *coli* and *K*. *pneumoniae* isolates that phenotypically expressed ESBLs in Tamale Teaching Hospital, Ghana (April—June 2015).

Resistance genes	Number of positive genotypes
TEM only	4 (6.5%)
SHV only	2 (3.2%)
CTX-M 1 only	11 (17.7%)
CTX-M 2 only	0 (0.0%)
CTX-M 9 only	0 (0.0%)
TEM + SHV	2 (3.2%)
TEM + CTX-M-1	29 (46.8%)
SHV + CTX-M-1	2 (3.2%)
TEM + SHV + CTX-M-1	4 (6.5%)
ESBL expressing isolates without identifiable ESBL genes	8 (12.9%)
**Total**	**62 (44.3%)**
Total TEM	39 (62.9%)
Total SHV	10 (16.1%)
Total CTX-M-1	46 (74.2%)

TEM-Temoniera, SHV- Sulfhydryl variable, CTX-M- Cefotaximase.

## Discussion

In this study of ESBLs in clinical isolates of *E*. *coli* and *K*. *pneumoniae* from patients attending TTH between April and June 2015, we had the following key findings: 1) there was a relatively high prevalence of ESBL during a three-month period in TTH; 2) there was a significant difference in AMR between ESBL-producers and non-ESBL-producers; and 3) the most common gene expressed among the isolates was *Bla*_CTX-M-1_.

In the present study, we report a relatively high prevalence of ESBL (44%) during a three-month period in TTH, Ghana. The proportion of ESBLs reported is higher compared to that reported in Tanzania (15%) [21] and Nigeria (20%) [22]. The prevalence recorded in this study is comparable to that documented in Nigeria (40%) [23] and KBTH (49.3%) Accra, Ghana [24] but lower than the prevalence reported from KATH (57.8%) Kumasi, Ghana [13]. The difference in percentages could probably be due to the differences in sample size at the various study sites. While the present study used a sample size greater than that which was reported in Nigeria, [22] the sample size was lower when compared to the study reported in Ghana [13]. Additionally, the high prevalence could be attributed to the ESBL menace existing in our societies and the lack of awareness may have increased the burden.

The general antimicrobial susceptibility pattern in the present study showed an overall high rate of resistance among the antibiotics used. Significant differences in AMR between ESBL producers and non-ESBL producers were observed for cefuroxime, gentamicin and amikacin similar to a study in Bangladesh [25]. The observed high prevalence of resistance to aminoglycosides in our study may be due to a number of mechanisms such as: (1) enzymatic modification and inactivation of the aminoglycosides, mediated by aminoglycoside acetyltransferases, nucleotidyltransferases, or phosphotransferases; (2) increased efflux; (3) decreased permeability; and (4) modifications of the 30S ribosomal subunit that interferes with binding of the aminoglycosides [26]. This may explain why both gentamicin and amikacin had high resistance rates of 24% and 18% respectively as observed in the study. Ghana has a high rate of aminoglycoside consumption. This is evident from both single-site [27, 28] and multi-center antibiotic use surveys [29, 30], which often list aminoglycosides among the top 8 most prescribed antibiotics in the country. Data from a six-year study at a secondary healthcare facility in Ghana further confirms this trend [31]. The study found that gentamicin and amikacin, the two most common aminoglycosides, were prescribed at a rate of 13.1 daily defined dose per 100 patients over six years, with no significant fluctuation in consumption from 2016 to 2021.

The high antibiotic resistance levels recorded in the present study (in which the isolates investigated were collected nearly eight years ago) are similar to those observed among Enterobacterales in several other studies conducted in Ghana, both older [11, 13, 24], and newer ones [32–37]. That the high AMR levels observed in this study are similar to those in these newer studies [32–37] suggests that the AMR situation in the country has been sustained, or possibly been exacerbated over the period, and could probably get worse. A meta-analysis may be needed to quantify the dynamics in the AMR problem in the country in the last decade or two; this could be done in other regions noted as hotspots for AMR occurrence. The findings of a study by Hackman and colleagues [38] with a high resistance of non-ESBL-producing bacterial isolates to ampicillin and tetracycline are confirmed in this study. In the present study, however, all the isolates were susceptible to meropenem. Meropenem maintains high efficacy against multidrug pathogens in Ghana [35, 39]. Meropenem is a more expensive antibiotic, and reserved by the Ghana Treatment Guidelines as a last resort for serious infections. Moreover, meropenem administration is parenteral and is less likely to be abused. This limited use makes it less susceptible to misuse and overuse, thereby protecting its effectiveness against highly resistant pathogens. While meropenem has been available in Ghana since 2002, its relatively short market presence compared to aminoglycosides contributes to its lower consumption rate. However, long-term use of meropenem can still lead to the selection of resistant strains.

In the present study, the majority (74%) of the isolates expressed *bla*_CTX-M-1_ genes. Similar findings were reported in a study in Pakistan, where 93% of isolates from a tertiary care centre were positive for CTX-M-1 genes [40]. Similar conclusions have been reported from other studies in India [41] and Iran [42]. *Bla*_CTX-M-1_ genes commonly found in *E*. *coli* isolates are increasingly being reported, [43] and the level of resistance conferred by these enzymes to cefotaxime and ceftazidime is high [44]. *The propagation of the highly conjugative bla*_CTX-M-1_ plasmids to pathogenic bacteria endangers human health [45]. From a One-Health perspective, the dominance of blaCTX-M-1 ESBL genes among the clinical isolates could suggests a potential link to animal sources [46, 47]. However, our inability to assess animal contact during the study precludes us from definitively establishing this connection. Notably, ESBL-producing bacteria harboring blaCTX-M-1 genes have been reported from diverse sources in Ghana, including food and farm animals and their settings [48, 49], patients [50, 51], healthcare facility settings [52], as well as potentially healthy community individuals [53] and their environment [54, 55].

The predominance of these enzymes among the study isolates may explain the widespread AMR levels among the isolates. The spread of these resistant isolates could have occurred via hospital cross infections, improper hand hygiene practices especially after visiting the lavatory, improper use of disinfectants and overcrowding in communities [56]. Among the ESBL expressing isolates, none expressed genes for CTX-M 2 and CTX-M 9. Also, ESBL expressing isolates without identifiable ESBL genes were approximately 13%. This observation suggests that the isolates could possess other ESBL enzyme types besides TEM, SHV and CTX-M-1 genes which were not sought for in the present study. None of the bacterial isolates were resistant to meropenem indicating its relevance in treating these emerging CTX-M-1 group positive bacteria.

Often, seriously ill patients have a risk of developing infections caused by ESBL-producing organisms. This is due to prolonged hospital stays and the use of invasive medical devices (urinary catheters, endotracheal tubes and central venous lines) [57]. Patients who reported that their previous treatment with antibiotics prescribed by a physician were more likely to be infected with ESBL-producing organisms. Patients who also completed their previous medication were found to have higher odds of infection with ESBL-producing enterobacteria. All participants in our study had received antibiotics within the past three months, highlighting the high prevalence of antibiotic use in Ghana [29, 30]. However, the specific antibiotics and treatment duration was unclear. This high level of antibiotic use in Ghana, particularly third-generation cephalosporins, may contribute to intestinal carriage and subsequent infections by antibiotic-resistant pathogens, especially those producing ESBLs. Heavy antibiotic use has been reported as a factor for acquisition of ESBL-producing enterobacteria [58, 59]. However, in the present study, this risk factor could not be evaluated, as the variable was not part of the laboratory data of the hospital studied.

The findings of this study should be interpreted with consideration of certain technical limitations. The study was conducted at a single hospital in Ghana over a three-month period, limiting the diversity of the patient population. This restricted scope may affect the generalizability of the findings to other regions or time periods. Additionally, the study lacked sufficient information on the antibiotic treatments received by patients and specific patient characteristics that could impact the development of ESBL infections. The questionnaire interview aspect of the study also imposes certain limitations in terms of recall bias on quality of data for the risk factor analysis. This lack of data prevented a comprehensive evaluation of potential risk factors, such as the duration of antibiotic use, which could provide a more thorough understanding of the factors contributing to ESBL infections. It is also worth noting that a wide range of other ESBL genes were not detected in this investigation. These include PER, IMP, and GES, which were not tested for because PCR was only carried out for some of the more common ESBL genes. The exclusion of these genes could potentially underestimate the diversity of ESBL genes within the Ghanaian community. Despite these limitations, our findings suggest a high prevalence of ESBL producers among the Enterobacterales in Ghana.

## Conclusion

We report a relatively high proportion of ESBL producers among *E*. *coli* and *K*. *pneumoniae* in the Northern part of Ghana, although the study was conducted at a single hospital over a three-month period, limiting the diversity of the patient population. Approximately six percent of ESBL-producing isolates harbored all three genes (*Bla*_TEM_, *Bla*_SHV_ and *Bla*_CTX-M-1_). The judicious use of antibiotics, especially meropenem, for improved human wellbeing should be urgently promoted in Ghana. To ensure rational empirical therapy, a reduction in antimicrobial abuse and not compromising on patients’ health, there is an urgent need for routine laboratory screening for ESBL-producing isolates. Proper antimicrobial administration to buttress the usefulness of active hospital surveillance programs for drug resistant bacteria is also warranted.

## Supporting information

S1 Raw image (PDF)
