# Supplementary figures and images for: Extended-spectrum beta-lactamases in clinical isolates of Escherichia coli and Klebsiella pneumoniae recovered from patients at the Tamale Teaching Hospital, Ghana
Source: PLoS One. 2024 Apr 5;19(4):e0300596. doi: 10.1371/journal.pone.0300596 (PMC10997077; doi:10.1371/journal.pone.0300596)

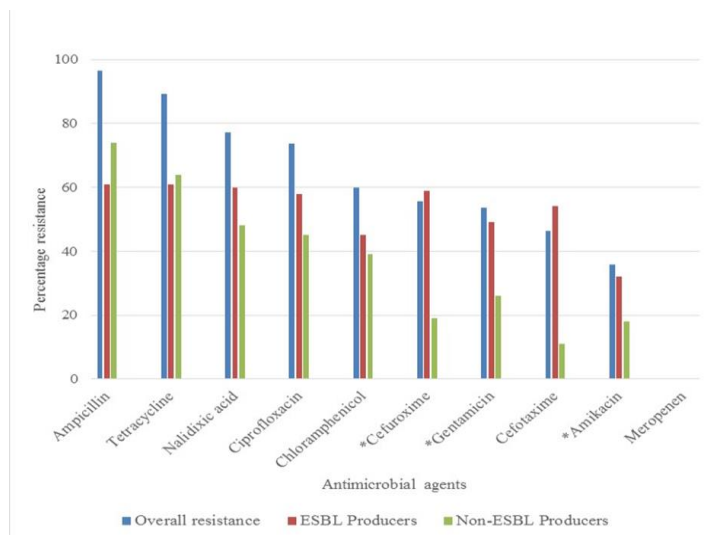

**Fig 1.**

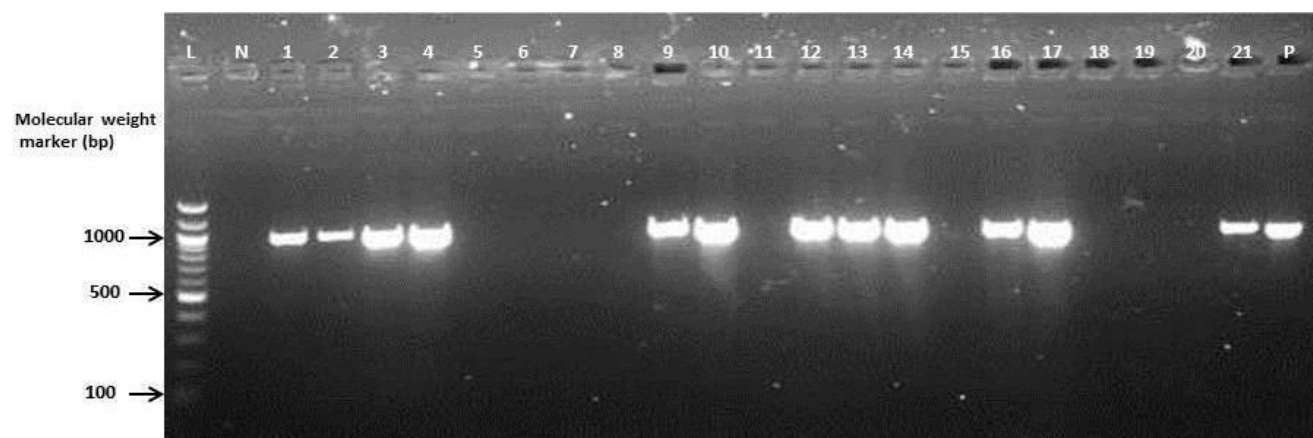

Fig 2.

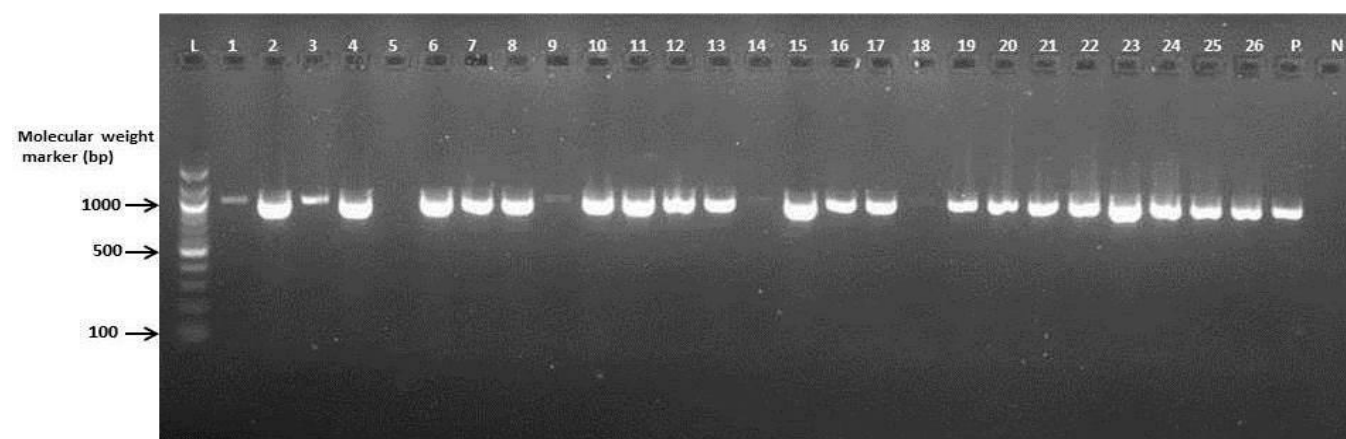

Fig 3.

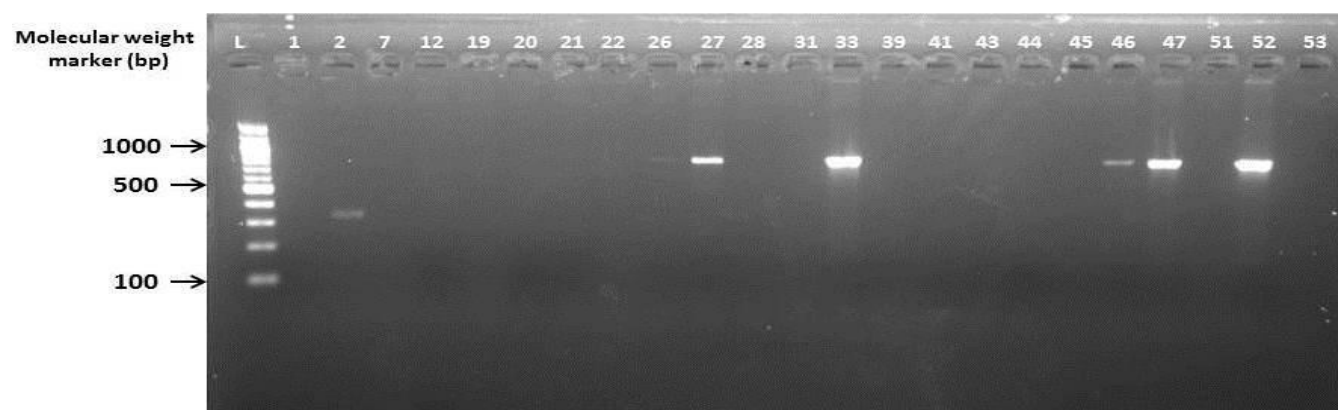

Fig 4.

Supplement: S1 Raw image — (PDF) [file pone.0300596.s001.pdf]
